# Perceived stress and bruxism in university students

**DOI:** 10.1186/s13104-016-2311-0

**Published:** 2016-12-21

**Authors:** Pierpaolo Cavallo, Luna Carpinelli, Giulia Savarese

**Affiliations:** 1 Department of Physics, University of Salerno, Via Giovanni Paolo II, 132, 84084 Fisciano, SA Italy; 2 Center of Psychological Counseling for Students, University of Salerno, Via Allende, 84081 Baronissi, SA Italy; 3 Department of Medicine and Surgery, University of Salerno, Via Allende, 84081 Baronissi, SA Italy

**Keywords:** Perceived stress, Bruxism, University, Student health, PSS-10

## Abstract

**Background:**

Many studies have shown the correlation between bruxism and stress that affects the quality of life of university students. The present study highlights this correlation—for the first time—in a group of university students in Italy.

**Methods:**

We have investigated the prevalence of awake and asleep bruxism and its correlation with perceived stress in a group of 278 Italian undergraduate students (117 M). A self report questionnaire was constructed using a socio-demographic test, the Perceived Stress Scale (PSS) and the item n. 8 of the Fonseca Questionnaire for presence of bruxism.

**Results:**

The perceived stress score using PSS-10 scale was 32.2 (SD 4.6, 95% CL 31.6–32.7) for all the subjects, with significant gender difference: M = 31.2 and F = 32.9 (P = 0.0019). The prevalence for awake bruxism was 37.9% (F = 40.8%; M = 34.2%,), while for sleep bruxism was 31.8% (F = 33.3%; M = 29.1%), both without significant gender difference. A positive correlation, with significant concordance and dependence, between stress score and awake bruxism was present for male students only.

**Conclusions:**

University students showed higher bruxism and stress levels compared to the general population, with higher stress for females, but, even if female students show higher stress, a correlation between stress and bruxism exists only for male gender. Further studies should be performed.

## Background

Stress is a universally used and well known word, in psychological, social, professional and school settings: the words “eustress”, in a positive connotation, and “distress”, in a negative one, describe the positive and negative aspects of stress in a person’s life.

University students may undergo an undue amount of stress, with negative outcomes [1] in terms of academic results and personal, emotional or health, consequences. Moreover, stress can be experienced at different time periods [2], not only during university life, but also before, during the transition from undergraduate to professional level, and after, during the transition to the life work.

Sources of stress in University students [3] include academic work, personal situations, environment, time, and economic circumstances.

One of the stress manifestations is bruxism, or gnashing and grinding of the teeth occurring without a functional purpose, variably frequent in the general population [4].

There has been an increase of bruxism among students in higher education, with epidemiological studies showing a prevalence rate growing from 5% in 1966 to 22% in 2002 [5], as stress prevalence showed to do in the same population [6].

Theories about the origin of bruxism [4] have hypothesized different types of factors: peripheral, connected to teeth occlusion interferences, central, connected to neurotransmission from brain to chewing muscles and psychosocial, associated to stress. According to current literature, stress plays an important role in the pathogenesis of bruxism, and consequently bruxism, with it being a clinical symptom that could be monitored in a relatively easy way and a potentially useful indicator of stress.

The number of studies on the relationship between bruxism and stress in university students has increased in recent years: the university setting, with its transitional nature, commitment and challenges [7–9], can be a time in which students can either learn to cope with it or remain unaware of its presence and become prone to its negative effects.

Thus, the prevalence of bruxism, and its correlation with perceived stress in a group of Italian University students was investigated. This is the first study investigating the presence of perceived stress in correlation with bruxism in Italian university students.

## Methods

The study aimed to investigate:

(i) the prevalence and intensity of bruxism and perceived stress in Italian university students;
(ii) the correlation between bruxism and perceived stress;
(iii) the presence of gender- and/or personal characteristics-related differences in correlation between bruxism and perceived stress.

The participants were a sample of 278 undergraduate students, all of Italian nationality and Caucasian race, studying at the Department of Science at the University of Salerno, Italy.

The total number of students in the Department was 1170, and our sample represented the 23.7% of all students, with an homogeneous distribution between the courses.

The study was conducted according to the guidelines of the Declaration of Helsinki, and was completely anonymous.

The study protocol was evaluated for Ethical Committee approval at University of Salerno, Italy. Given its anonymousness, voluntariness of participation, absence of risk or burden, sponsors, conflicts of interest and incentives for the responding subjects, no approval was considered necessary.

Information on study protocol were given, and informed consent was obtained, before administration of an anonymous questionnaire.

To improve anonymousness, the questionnaire sheets were randomly numbered and collated for data analysis only after they had all been gathered, at the end of the day, to prevent from any possibility of identification of the responding subject by means of the sheet sequence.

The questionnaire was administered during the breaks between lessons, in the middle of the semester and completed by all the participants without any difficulties in 15 min or less. The moment was chosen since it was far from any seasonal holidays and stressful periods, usually concentrated either at the beginning, due to novelty to challenge, or at the end of the semester, due to exams.

The questionnaire consisted of three sections: (i) socio-demographic test; (ii) the 10-item Perceived Stress Scale (PSS); (iii) a section about the presence of bruxism.

The demographic section asked the respondent’s age and gender, plus a number of social and behavioral questions: living with own family, practicing regular exercise, smoking cigarettes, consuming alcoholic beverages, experiencing abuse drugs.

The PSS-10 [10, 11] measures self-reported stress and was used because of its established validity and reliability; it includes 10 questions, with answers ranked using a 5-point Likert scale, and assesses stressful experiences and responses to stress over the previous 4 weeks. Questions that relate negative events or responses are scored in a reverse manner. Scores range from 0 to 56, with higher scores indicating higher levels of perceived stress.

Bruxism can be investigated with the Fonseca Questionnaire [12], an instrument designed to assess the prevalence and severity of temporomandibular disorders. Item 8 “Do you clench or grind your teeth?” was used to assess the presence of bruxism, and the question was proposed in two different forms: (i) “Do you clench or grind your teeth when you are awake?”, and (ii) “Do you clench or grind your teeth when you are asleep?”. The answers were scored on a Likert-type scale, ranging from “never” to “always”, from 1 to 5.

Descriptive statistics were obtained and the comparison between the variables was performed by one-way ANOVA. The correlation was measured with Kendall’s tau-b method, that enables to measure correlation between variables with different scales, as Bruxism was ordinal and stress was an interval. This method is similar to the well-known Spearman’s methods for rank correlation, but works better because is able to reflect the strength of the relationship between the variables.

The data were analyzed using the statistical package StatsDirect Version 3 (StatsDirect Ltd.).

## Results

The genders were well balanced in the group, with 117 (42.7%) male subjects and the mean age for all was 23.7 years, without statistical significance between M and F.

The personal characteristics are presented in Table 1, along with their frequencies for gender and P value for difference.

**Table 1:** Personal characteristics

Parameter	All subjects %	M %	F %	P
Living with one’s family	63.1	78.6	51.5	<0.001**
Regular exercise	61.7	84.1	44.5	<0.001**
Smokes cigarettes	19.7	13.6	24.2	0.023
Consumed alcoholic beverages	36.1	39.3	33.7	NS
Experienced drugs	16.4	17.0	15.9	0.01*

*NS* not significant

*P < 0.05 is statistically significant; **P < 0.001 is statistically highly significant

PSS-10 showed a good reliability, with Cronbach Alpha = 0.78; the mean score was 32.2 (SD 4.6, 95% CL 31.6–32.7), while the results by gender were M = 31.2 and F = 32.9, with the latter being significantly higher (P = 0.0019**).

The prevalence of bruxism was measured considering all answers different from “never” as affirmative.

The prevalence of awake bruxism (BRUX1) was 37.9% in the whole sample, with gender prevalence for F = 40.8% and M = 34.2%, without any statistically significant difference (P = 0.082).

The prevalence of sleep bruxism (BRUX2) was 31.8% in the whole sample, with gender prevalence for F = 33.3% and M = 29.1%, without any statistically significant difference (P = 0.369).

We investigated the differences in PSS-10 and Bruxism (BRUX1 and BRUX2) scores according to personal characteristics and gender; the results are presented in Table 2.

**Table 2:** Mean scores for perceived stress by personal characteristics, bruxism and gender

Parameter	PSS-10 M	PSS-10 F	P	BRUX1 M	BRUX1 F	P	BRUX2 M	BRUX2 F	P
Lives with own family (M = 106 F = 138)	30.8	33.3	0.03*	1.5	1.87	0.03*	1.52	1.73	NS
Lives out of family (M = 13; F = 20)	31.3	32.6	0.03*	2.2	1.7	NS	1.91	1.93	NS
Regular exercise (M = 75; F = 69)	31.2	32.6	NS	1.62	1.77	NS	1.52	1.69	NS
No regular exercise (M = 42; F = 79)	30.7	33.2	0.02*	1.77	1.83	NS	1.51	1.72	NS
Smokes cigarettes (M = 45; F = 39)	29.3	33.2	0.04*	1.31	1.94	NS	1.5	1.92	NS
Non smoker (M = 133; F = 118)	31.5	32.8	0.01*	1.18	1.74	<0.001**	1.52	1.64	NS
Consumed alcoholics (M = 84; F = 60)	30.7	33.5	0.01*	1.73	2.09	NS	1.5	2.05	0,01*
No alcoholics (M = 52; F = 97)	31.4	32.6	NS	1.59	1.66	NS	1.57	1.5	NS
Experienced drugs (M = 36; F = 26)	30.5	32.6	NS	2.15	1.84	NS	1.75	1.8	NS
No drugs (M = 81; F = 131)	31.3	33.0	0.002**	1.54	1.80	NS	1.41	1.69	NS

PSS-10 = perceived stress score, 10-item scale

*BRUX1* bruxism awake; *BRUX2* sleep bruxism; *NS* not significant

*P < 0.05 is statistically significant; **P < 0.001 is statistically highly significant

Some statistically significant gender differences were highlighted according to the studied variables.

For the “living in family” variable, the female subjects always had higher stress levels, and those living with their own family also had higher BRUX1 (awake bruxism) levels.

For the “regular exercise” variable, the female subjects not taking any regular exercise showed higher stress levels, while for the “smoking” variable, the female subjects once again showed higher stress levels, the non-smokers also had higher BRUX1 levels; for the “alcohol” variable, the female subjects consuming alcohol had higher stress levels; finally, for the “drug” variable, the female subjects who had no experience with drugs showed higher stress levels.

The BRUX2 scale (sleep bruxism) showed higher levels for students living out of family, with no gender differences; only one remarkable gender difference for BRUX2 was shown in the group of alcoholics consumers, in which F gender showed a significantly higher score (2.05 vs. 1.5, P = 0.01*).

The correlation between bruxism and perceived stress was also investigated according to characteristics and gender; Kendall’s analysis was used, since it measures the correlation but also shows the possible presence of a dependence between the variables; the results are presented in Table 3.

**Table 3:** Correlation between bruxism and perceived stress by personal characteristics and gender, Kendall’s Tau method

Parameter	PSS-10 vs. BRUX1 M	PSS-10 vs. BRUX2 M	PSS-10 vs. BRUX1 F	PSS-10 vs. BRUX2 F
Lives with own family (M = 106; F = 138)	0.17 NS	0.01 NS	0.06 NS	0.15 NS
Lives out of family (M = 13; F = 20)	0.976 Conc. 0.009** Dep. 0.001**	0.21 NS	−0.01 NS	−0.19 NS
Regular exercise (M = 75; F = 69)	0.21 Conc. 0.01* Dep. 0.03*	0.09 NS	0.08 NS	−0.15 NS
No regular exercise (M = 42; F = 79)	0.25 Conc. 0.01* Dep. 0.02*	−0.24 Disc. 0.02* Dep. 0.05*	0.02 NS	0.04 NS
Smokes cigarettes (M = 45; F = 39)	−0.09 NS	0.01 NS	−0.05 NS	−0.10 NS
Non smoker (M = 133; F = 118)	0.917 Conc. 0004** Dep. 0009**	−0.03 NS	0.09 NS	−0.02 NS
Consumed alcoholics (M = 84; F = 60)	0.29 Conc. 0,01* Dep. 0.02*	0.01 NS	0.16 Conc. 0.04* Dep. 0.08 NS	−0.06 NS
No alcoholics (M = 52; F = 97)	0.19 Conc. 0.03 Dep. 0.06 NS	−0.1 NS	0,15 Conc. 0.04* Dep. 0.09 NS	−0.02 NS
Experienced drugs (M = 36; F = 26)	0.17 Conc. 0.03* Dep. 0.06 NS	−0.03 NS	0.09 NS	0.03 NS
No drugs (M = 81; F = 131)	0.18 Conc. 0.02* Dep. 0.04*	−0.02 NS	0.04 NS	−0.05 NS

Tau-b value is reported, P is reported if significant

*Conc* concordance; *Disc* discordance; *Dep* dependence; *NS* not significant

*P < 0.05 is statistically significant; **P < 0.001 is statistically highly significant

The Kendall’s rank analysis showed the presence of concordance between the stress and the BRUX1 (awake) scores in the male subjects for nearly all the parameters studied. The strongest concordance values, with the lowest P values and a highly significant dependence, were found for “not living with own family” as well as for the “non-smokers” subgroup.

For BRUX2 (asleep) scores, only one group, namely the “no regular exercise” of M gender showed a negative correlation between stress and bruxism, with a statistical significance for Discordance and Dependence in this group.

## Discussion

Generally, it is possible to affirm that the higher stress levels of students compared to the general population data could be related to the commitment and challenges of their “job”, and is consistent with previous literature [7–9].

In addition, the finding of a higher bruxism prevalence for students in respect of the general population data appeared to be consistent with current literature. Even if there is a limited number of studies, a recent review has shown [13] a prevalence ranging from 8 to 31.4%., and the highest prevalence of the above mentioned review was found in the Italian general population [14]; moreover, recent literature has shown levels of bruxism up to 83% in dentistry students [15].

The main findings will now be discussed separately, with them being:

(i) the correlation between stress and bruxism in university students;
(ii) the presence of a gender difference in stress for university students;
(iii) the presence of a gender related correlation between stress and bruxism only in male university students.

The correlation between stress and bruxism is reported in current literature: for example, this finding was reported in a previous study on occupational stress [16], and, more specifically, in university students stress can induce neuromuscular alterations in the mouth and jaw, increasing the general prevalence of temporo-mandibular disorders [17].

Bruxing subjects differ from healthy individuals in the presence of stress sensitivity [13], with daytime teeth clenching (BRUX1) significantly being explained by experienced stress [18], while sleep bruxism (BRUX2) is considered a sleep movement disorder of central origin [13].

In terms of studies in the specific college/university setting, an association between bruxism and stress has been shown [5, 19–22], and literature reports an increase in the incidence of self-reported nocturnal bruxism in college students over the last decades [5] with recent literature reporting also very high values, as we mentioned above [15].

The presence of a gender difference in stress for university students is also consistent with current literature: the majority of the studies reported stress as being higher for female students [16, 23–25]. In our study, the higher prevalence of stress for female subjects living in their own family could be explained by the higher psychological pressure and expectations, and could also plausibly connected with the higher stress levels for female subjects who do not smoke and have not experienced drugs, according to the possible action of the former as a stress reliever and the latter as an escape from reality.

Previous studies have shown that bruxism in the general population is predominant among females [26], and in students there is also a higher prevalence for females [16, 27–30], which we have confirmed in our study, either for BRUX1 and BRUX2, even if the gender difference was not statistically significant.

The presence of a gender related correlation between stress and daytime bruxism (BRUX1) in M gender subjects could be the most innovative part of the research and may be useful to stimulate further studies.

In fact, a correlation between stress and BRUX1 in the male subjects was found, with the higher values being for those living away from their families and for the non-smokers. This could be explained by the higher psychological pressure on males who have to face the challenge of university life as well as manage living on their own, while it is the opposite for females, who experience more stress when living with their own families.

Being a non-smoker may play a similar role between the genders, but with different outcomes: non-smoking females have higher stress levels, but no correlation with bruxism, while non-smoking males have lower stress levels but show stress under the form of awake bruxism.

On the contrary, sleep bruxism (BRUX2) did not show significant correlation with stress, in accordance with the different etiology of these two disorders.

A possible explanation could be related to the pathophysiological factors [4] modulating the bruxism: it is a multidimensional phenomenon, [31] mainly regulated centrally [32] and associated to perceived stress [33].

On these bases, we hypothesize a possible cascade.

It could start from the psychosocial factors, tied to stress, and then could act via central factors, tied to neurotransmission from the brain to the chewing muscles.

These could transfer the burden of stress on the teeth through peripheral factors, and these could finally cause the occlusion interferences.

The fact that in our study BRUX1 does correlate with stress while BRUX2 does not, may be considered a further demonstration of the etiological difference between these two conditions.

## Limitations

This study has several limitations: it was only a cross-sectional study, assessing bruxism through a questionnaire, since bruxism was self-reported and not confirmed by dental examination; the sample was highly specific, and, finally, there was a slight difference in sample size by gender, even if the age was homogeneous.

Moreover, the differences in culture and life experience between university settings in different nations suggest prudence in generalizing the findings, even if they could possibly stimulate further studies.

## Conclusions

Notwithstanding the limitations, it is possible to affirm that university students show a higher awake bruxism and stress levels in relation to the general population, that a correlation exists between awake bruxism and stress, and that there is a gender difference for the presence of stress.

It is also possible to state that the correlation between stress and awake bruxism, is gender-related, being present only in male university students.

### Research agenda

The gender differences may play a role in the levels of stress and the presence of bruxism in university students, and should be taken into account for future research.

We suggest, as a research agenda for the future, that further studies may be performed to distinguish sleep bruxism and awake bruxism, considering also the respective etiology, assess the relationship of bruxism with stressors, possibly comparing different populations to account for the effects of different socio-cultural and university organization settings.
